# A comparison of the cumulative incidence and early risk factors for psychotic disorder in young adults in the Northern Finland Birth Cohorts 1966 and 1986

**DOI:** 10.1017/S2045796016000123

**Published:** 2016-03-28

**Authors:** S. Filatova, R. Marttila, H. Koivumaa-Honkanen, T. Nordström, J. Veijola, P. Mäki, G. M. Khandaker, M. Isohanni, E. Jääskeläinen, K. Moilanen, J. Miettunen

**Affiliations:** 1 Center for Life Course Health Research, University of Oulu, Oulu, Finland; 2 Medical Research Center Oulu, Oulu University Hospital and University of Oulu, Oulu, Finland; 3 Institute of Clinical Medicine (Psychiatry), University of Eastern Finland, Kuopio, Finland; 4 Departments of Psychiatry, Kuopio University Hospital, Kuopio; 5 Departments of Psychiatry, South-Savonia Hospital District, Mikkeli; 6 Departments of Psychiatry, North Karelia Central Hospital, Joensuu; 7 Departments of Psychiatry, SOSTERI, Savonlinna; 8 Departments of Psychiatry, SOTE, Iisalmi; 9 Departments of Psychiatry, Lapland Hospital District, Rovaniemi, Finland; 10 Department of Psychiatry, Center of Clinical Neuroscience, University of Oulu, Oulu, Finland; 11 Department of Psychiatry, Oulu University Hospital, Oulu, Finland; 12 Department of Psychiatry, The Middle Ostrobothnia Central Hospital, Kiuru, Finland; 13 Department of Psychiatry, Mental Health Services, Joint Municipal Authority of Wellbeing in Raahe District, Finland; 14 Department of Psychiatry, Mental Health Services, Basic Health Care District of Kallio, Finland; 15 Department of Psychiatry, Visala Hospital, Ylivieska, the Northern Ostrobothnia Hospital District, Finland; 16 Department of Psychiatry, University of Cambridge, Cambridge, UK; 17 Cambridgeshire and Peterborough NHS Foundation Trust, Cambridge, UK; 18 Unit of Primary Health Care, Oulu University Hospital, Oulu, Finland; 19 Department of Psychiatry, Länsi-Pohja Healthcare District, Finland

**Keywords:** Cumulative incidence, early risk factors, psychosis, schizophrenia

## Abstract

**Aims.:**

Few studies have compared time trends for the incidence of psychosis. To date, the results have been inconsistent, showing a decline, an increase or no significant change. As far as we know, no studies explored changes in prevalence of early risk factors. The aim of this study was to investigate differences in early risk factors and cumulative incidences of psychosis by type of psychosis in two comparable birth cohorts.

**Methods.:**

The Northern Finland Birth cohorts (NFBCs) 1966 (*N* = 12 058) and 1986 (*N* = 9432) are prospective general population-based cohorts with the children followed since mother's mid-pregnancy. The data for psychoses, i.e. schizophrenia (narrow, spectrum), bipolar disorder with psychotic features, major depressive episode with psychotic features, brief psychosis and other psychoses (ICD 8–10) were collected from nationwide registers including both inpatients and outpatients. The data on early risk factors including sex and place of birth of the offspring, parental age and psychosis, maternal education at birth were prospectively collected from the population registers. The follow-up reached until the age of 27 years.

**Results.:**

An increase in the cumulative incidence of all psychoses was seen (1.01% in NFBC 1966 *v*. 1.90% in NFBC 1986; *p* < 0.001), which was due to an increase in diagnosed affective and other psychoses. Earlier onset of cases and relatively more psychoses in women were observed in the NFBC 1986. Changes in prevalence of potential early risk factors were identified, but only parental psychosis was a significant predictor in both cohorts (hazard ratios ≥3.0; 95% CI 1.86–4.88). The difference in psychosis incidence was not dependent on changes in prevalence of studied early risk factors.

**Conclusions.:**

Surprisingly, increase in the cumulative incidence of psychosis and also changes in the types of psychoses were found between two birth cohorts 20 years apart. The observed differences could be due to real changes in incidence or they can be attributable to changes in diagnostic practices, or to early psychosis detection and treatment.

## Introduction

Time trends in the incidence of schizophrenia and other psychoses have been challenging to investigate due to the lack of suitably large and comparable samples from different time periods. Other challenges include differences and changes in population structure, diagnostic criteria, risk factors, health care organisation, and biases in data recording (Kendell *et al*. 1993). Studies have not shown large difference in incidence rates across countries worldwide (Jablensky, 2000). In a systematic review of studies from 33 countries, the median value of the incidence of schizophrenia was 15.2 (7.7–43.0) per 100 000 (McGrath *et al*. 2004).

Results of previous incidence studies of psychosis over time in high-income countries since 1960s have been inconsistent, some reporting a decline (Takei *et al*. 1996; Brewin *et al*. 1997; Suvisaari *et al*. 1999; Ösby *et al*. 2001) increase (Häfner & an der Heiden 1986; Bamrah *et al*. 1991) or no change in incidence (Allardyce *et al*. 2000; Kirkbride *et al*. 2009). Studies from the Nottingham, England reported greater variety of psychotic diagnoses in cohort 1992–1994 compared with 1978–1980 (Brewin *et al*. 1997) and increase in other non-affective psychoses (away from schizophrenia) over three time periods (1978–1980, 1993–1995 and 1997–1999) (Kirkbride *et al*. 2009). A meta-analysis suggested the pooled annual incidence of all psychoses in England over the period 1950–2009 was 31.7 (95% CI 24.6–40.9) per 100 000 person-years and 15.2 (95% CI 11.9–19.5) for schizophrenia (Kirkbride *et al*. 2012). The results of these studies support that incidences of schizophrenia do not vary significantly, but estimates for all psychoses vary over time, indicating inconsistency between studies on time trends of schizophrenia (McGrath *et al*. 2004; Kirkbride *et al*. 2009).

In Finland the lifetime prevalence of any psychosis varies regionally from 2.2 to 4.6%, with the highest prevalence in Northern Finland. The differences have been explained by area-related environmental factors (e.g. more underweight newborns in rural Eastern and Northern regions) and genetic predisposition (Perälä *et al*. 2008). Suvisaari *et al*. (1999) identified a decline in the incidence of schizophrenia in Finland in the period 1954–1965 and Haukka *et al*. (2001) found that urban birth increased incidence in cohorts born since 1955.

In the beginning of the 1990s in Finland new Mental Health Act led to significant changes and resulted in integration of mental health with somatic health and social services, the decentralisation of the financing and the deinstitutionalisation process. A reduction of psychiatric beds in hospitals from 20 000 to 6000 during period of 20 years since 1980s was followed by development of out-patient care and community based mental health services. It has been claimed sometimes that outpatient care did not receive enough resources in that period. On the other hand, extra budget was allocated for the development of mental health services for young people (Lehtinen & Taipale, 2001). Furthermore, the diagnostic system of diseases in Finland has changed from ICD 8 (1968–1986) via ICD 9 (1987–1995) to ICD 10 (1996–2012).

Previous studies have identified a number of early risk factors for schizophrenia and psychosis such as male sex (Matheson *et al*. 2011), family history of schizophrenia (Gottesman, 1991) or other psychoses and severe mental disorders (Matheson *et al*. 2011), advanced paternal age (Miller *et al*. 2011), unwanted pregnancy (Myhrman *et al*. 1996) and maternal depression during pregnancy in offspring with parental psychosis (Mäki *et al*. 2010), obstetric complications (Matheson *et al*. 2011), low birth weight (Isohanni *et al*. 2006), infections in the central nervous system during childhood (Khandaker *et al*. 2014), stressful life events and childhood adversities (Varese *et al*. 2012), cannabis use later in life and urban residence (Matheson *et al*. 2014). A higher incidence of psychosis across the lifespan among males than females has been reported (Kleinhaus *et al*. 2011) and previous studies on gender differences in onset age focus primarily on schizophrenia rather than other psychoses (Gureje, 1991). Many risk factors are non-specific for schizophrenia and overlap with other psychotic and psychiatric disorders (Laursen *et al*. 2007), but this area requires further investigation (Laurens *et al*. 2015). Still, there seems to be lack of studies on consistency of early risk factors in the same geographical region over time.

Finland offers an ideal setting to explore time trends in the incidence of psychosis due to its high-quality health care registers, personal identity code use for each resident, a homogenous population, and a low migration rate (Miettunen, 2011). Furthermore, our data base, the two Northern Finland Birth Cohorts (NFBC 1966 and NFBC 1986) are unique, comparable and large general population cohorts based in the same geographical region with data collected prospectively since pregnancy.

The aim of the present study is to explore in two cohorts setup 20 years apart: (1) the cumulative incidence of psychoses and age of illness onset; (2) changes in type of diagnosis; and (3) changes in the early risk factors in order to be able to understand changes and determinants of the risk of psychosis over time.

## Methods

### Sample

The NFBC 1966 (*N* = 12 058) and 1986 (*N* = 9432) are prospective birth cohorts covering the two northernmost former provinces of Finland (i.e. Oulu and Lapland) with a population of 604 000 and 630 000 at their baseline data collection, respectively (Statistics Finland, 2015). The authors assert that all procedures contributing to this work comply with the ethical standards of the relevant national and institutional committees on human experimentation and with the Helsinki Declaration of 1975, as revised in 2008.

### Identification of psychoses

The data for psychoses were collected from several registers:

(1) Care Register for Health Care (CRHC);
(2) Finnish outpatient registers;
(3) Social Insurance Institution (SII) registers: reimbursable medicines, sick days, and disability pensions;
(4) Finnish Centre for Pensions: disability pensions.

For more details on the registers and time periods see Supplementary material 1. We included data only up to the age 27 years in both cohorts for study subjects as this was the average age at the last follow-up in NFBC 1986. Parental psychosis was identified through disability pensions registers since 1964, CRHC since 1972, specialised outpatient care data since 1998 and primary care outpatient care data since 2011 (Miettunen, 2011) until children's age 27, except sick days and reimbursable medicines which were not available for parents.

### Diagnostic criteria and age of illness onset

Psychoses were defined according to ICD 8 (1968–1986), ICD 9 (1987–1995) or ICD 10 (1996–2012). The diagnostic categories included are presented in Table 1. The diagnosis and age of onset were based on the first day of any psychosis treatment recorded in the registers. In case of disability pensions registers, the onset was defined as 1 year before the recorded date, since in Finland disability pension is allowed at the earliest 1 year after the onset of psychosis. The earliest age of onset was defined as a minimum 12 years old due to inconsistency in psychosis diagnosis in childhood (McKenna *et al*. 1994).

**Table 1.** Diagnostic categories of psychotic disorder based on ICD 8–10

Diagnosis	ICD-8 code (1968–1986)	ICD-9 code (1987–1995)	ICD-10 code (1996–2012)
Schizophrenia	295, 2954	295, 2954	F20
Schizophrenia spectrum			
Schizoaffective disorder	2957	2957	F25
Delusional disorder	297	297	F22
Bipolar disorder (with psychotic features)	2961–2969	2962E, 2963E, 2964E, 2967	F302, F312, F315
Major depressive disorder (with psychotic features)	2960, 2980	2961E	F323, F333
Brief psychosis	298 (except 2980)	2988	F23, F24
Other non-organic psychoses	299	2989	F28,F29

### Early risk factors

We investigated some of the risk factors that were reported to be associated with psychosis in previous reviews, if the comparable data were available for both NFBCs.

The following variables were used as predictors of psychosis: sex, diagnosis of psychosis in parents before the participant was 27 years of age (yes/no), paternal age (<25, 25–40, >40 years), maternal age at delivery (<20, 20–35, >35 years), place of residence at birth (urban/rural) and maternal education at baseline (basic, secondary, higher education). Urban residence was classified as born in the six largest cities in the region (Statistics Finland, 2015). The categorical variables for parental age and maternal education were created based on the previous NFBC studies (Keskinen *et al*. 2013).

Data on birth dates (cohort members and their parents), sex and parental psychosis were obtained from the national registers (cf. above). At baseline (i.e. at birth), information on place of residence and maternal education was collected from parents’ questionnaires.

### Statistical analysis

The chi-square test was used to compare categorical variables (sex, parental psychosis, maternal education, place of residence, maternal and paternal age) and student's *t*-test was used to compare the continuous variable (onset age of psychosis). Cox regression was applied in the univariate analyses to explore the relationships between the exposures and the outcome. We assumed that adjusting would not have given reliable estimates of hazard ratios (HRs) since most of the unadjusted HRs were non-significant and number of cases was relatively low. Association between changes in risk factors and incidence was studied with the pooled data (NFBCs 1966 and 1986) by cox regression with a cohort membership variable as a predictor and other risk factors as covariates in a multivariate analysis. Times of emigration and death were used as censoring points in analyses (information from the Population Register Centre). Cohort members with undefined outcome due to death and emigration before age of 12 (minimum age of onset) were excluded (*n* = 901 in NFBC 1966; *n* = 150 in NFBC 1986). Risk estimates were expressed as HRs with 95% confidence interval (95% CI). Interactions between cohort membership and risk factors (cohort*risk factor) were also studied using Cox regression. Data analyses were carried out using SPSS Statistics version 21, International Business Machines (IBM).

## Results

### Cumulative incidence and age of onset of psychosis

**Fig. 1.**
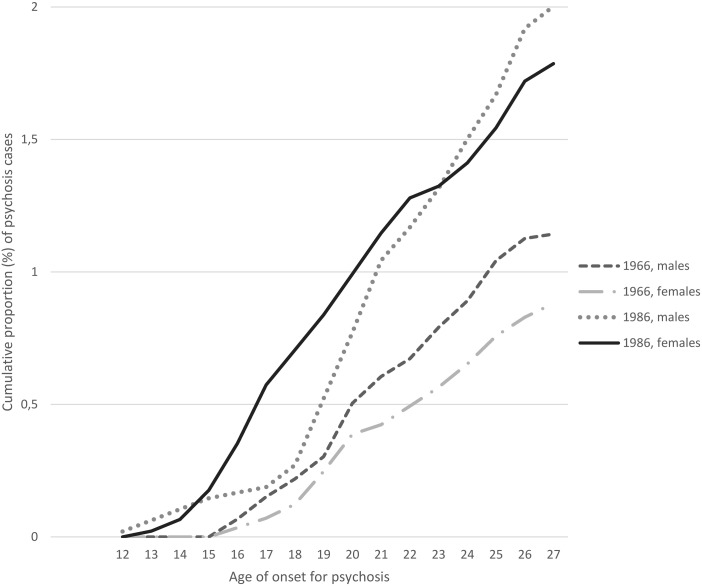
Cumulative incidences of psychoses among males and females in the NFBC 1966 and NFBC 1986.

The cumulative incidence of psychosis was lower in the NFBC 1966 than in the NFBC 1986 (1.01 *v*. 1.90%, *p* < 0.001) (Fig. 1). The mean, median and standard deviation (s.d.) of age of onset for psychosis did not differ between the total cohorts (*p* = 0.08) being 21.54, 21.45 (s.d.: 3.22) years in the NFBC 1966 and 20.94, 20.97 (s.d.: 3.76) years in the NFBC 1986, respectively. Among women, the NFBC 1986 had significantly earlier mean age of onset of psychosis than NFBC 1966 (i.e. 20.15 (3.88) *v*. 21.74 (3.36), *p* = 0.018). To investigate distinct trend (Fig. 1), men and women were compared until age of 18 years. Until that age women in the NFBC 1986 had a higher cumulative incidence of psychosis compared to men in the NFBC 1986 and women in the NFBC 1966 (*p* < 0.001). However, after age of 18 years the difference between males and females in NFBC 1986 was no longer significant (*p* = 0.166).

### Types of diagnosis

A shift in types of diagnosis between the two cohorts was found (Table 2). While the proportion of schizophrenia was quite similar (0.45/0.42%), more cases with schizophrenia spectrum, affective psychoses (bipolar disorder, major depression) and other psychoses (brief psychosis, other non-organic psychoses) were seen in the NFBC 1986. Among all psychotic cases, altogether 4% (*n* = 5) had bipolar disorder (psychotic) and 2% (*n* = 2) had major depression (psychotic) in the NFBC 1966, while the respective figures for NFBC 1986 were, 10% (*n* = 17) and 15% (*n* = 27) (in Table 2 the proportions are calculated by the total study sample).

**Table 2.** Cumulative incidence *(*%*)* of different psychotic disorders in the cohorts by the age of 27 years

	NFBC 1966 (*N* = 11 621)	NFBC 1986 (*N* = 9329)	
Diagnosis	*n* (%)	*n* (%)	*p*-value
Schizophrenia	53 (0.45%)	39 (0.42%)	0.751
Schizophrenia spectrum*	8 (0.06%)	11 (0.12%)	0.257
Bipolar disorder with psychotic features	5 (0.04%)	17 (0.18%)	0.002
Major depressive episode with psychotic features	2 (0.02%)	27 (0.29%)	*p* < 0.001
Brief psychosis	13 (0.11%)	20 (0.21%)	0.078
Other non-organic psychoses	37 (0.32%)	63 (0.68%)	*p* < 0.001
Total	118 (1.01%)	177 (1.90%)	*p* < 0.001

* Note: schizophrenia spectrum includes schizoaffective disorder and delusional disorder.

### Early risk factors

Socio-demographic characteristics and distribution of early risk factors of psychosis differed between the two cohorts. The NFBC 1966 had more subjects with a parental psychosis (6.4 *v*. 4.6%), fewer subjects with urban residence (30.5 *v*. 43.3%) and with mothers having at least a secondary education (22.7 *v*. 67.2%) compared to NFBC 1986 (Table 3). In addition, higher proportions of parents were 20–35 years old and fewer were <20 years old in the NFBC 1986 compared to the NFBC 1966.

**Table 3.** Distribution of early risk factors

Predictor	NFBC 1966, *n* (%)	NFBC 1986, *n* (%)	
Sample	*N* = 11 621	*N* = 9321	*p*-value
Sex			
Male	5949 (51.2%)	4794 (51.4%)	*p* = 0.778
Female	5672 (48.8%)	4535 (48.6%)	
Parental psychosis (until age 27)			*p* < 0.001
Yes	746 (6.4%)	430 (4.6%)	
No	10 875 (93.6%)	8899 (95.4%)	
Mother's education at birth			
Basic education (9 years)	8361 (73.2%)	2100 (26.5%)	*p* < 0.001
Secondary education	2591 (22.7%)	5334 (67.2%)	
Higher education	464 (4.1%)	503 (6.3%)	
Place of residence at birth			
Urban	3547 (30.5%)	3938 (43.3%)	*p* < 0.001
Rural	8074 (69.5%)	5158 (56.7%)	
Maternal age at birth (years)			
<20	1144 (9.9%)	394 (4.2%)	*p* < 0.001
20–35	8431 (73.2%)	7710 (82.6%)	
>35	1945 (16.9%)	1225 (13.1%)	
Paternal age at birth (years)			
<25	2405 (22.2%)	1568 (17.0%)	*p* < 0.001
25–40	7092 (65.6%)	7052 (76.4%)	
>40	1315 (12.2%)	614 (6.6%)	

Only parental psychosis was found to be a predictor of psychosis in both cohorts with about a threefold risk increase: HR_1966_ = 3.01 (95% CI 1.86–4.88); HR_1986_ = 2.99 (95% CI 1.91–4.67) (Table 4). There was no evidence for an interaction between cohort membership and any of the studied risk factors (i.e. sex, parental psychosis, baseline mother's education, place of residence and parental age) in the causation of psychosis (data not shown).

**Table 4.** Univariate analysis of early risk factors for psychosis in the cohorts presented in HR

	NFBC 1966 *N* = 11 621		NFBC 1986 *N* = 9321	
Predictor	*n*/*N* (%)	HR (95%CI)	*n*/*N* (%)	HR (95%CI)
Sex				
Male	68/118 (57.6%)	1.30 (0.90–1.87)	96/177 (54.2%)	1.12 (0.84–1.51)
Female	50/118 (42.4%)	1	81/177 (45.8%)	1
Parental psychosis (until age 27)				
No	98/118 (83.1%)	1	155/177 (87.6%)	1
Yes	20/118 (16.9%)	3.01 (1.86–4.88)	22/177 (12.4%)	2.99 (1.91–4.67)
Mother's education at birth				
Basic education	80/117 (68.4%)	0.77 (0.51–1.17)	41/143 (28.7%)	1.15 (0.79–1.66)
Secondary education	32/117 (27.4%)	1	91/143 (63.6%)	1
Higher education	5/117 (4.3%)	0.87 (0.34–2.23)	11/143 (7.7%)	1.29 (0.69–2.41)
Place of residence				
Urban	38/118 (32.2%)	1	82/171 (48.0%)	1
Rural	80/118 (67.8%)	1.08 (0.74–1.59)	89/171 (52.0%)	1.21 (0.90–1.64)
Maternal age				
<20	10/117 (8.5%)	0.89 (0.46–1.72)	9/177 (5.1%)	1.23 (0.63–2.41)
20–35	83/117 (70.9%)	1	143/177 (80.8%)	1
>35	24/117 (20.5%)	1.26 (0.80–1.98)	25/177 (14.1%)	1.01 (0.72–1.68)
Paternal age				
<25	19/112 (17.0%)	0.69 (0.42–1.14)	28/176 (15.9%)	0.94 (0.62–1.41)
25–40	81/112 (72.3%)	1	134/176 (76.1%)	1
>40	12/113 (10.7%)	0.80 (0.44–1.47)	14/176 (8.0%)	1.21 (0.70–2.10)

HR, hazard ratio; 95% CI, 95% confidence interval.

We explored association between changes in risk factors and incidence. The unadjusted HR for the cohort membership variable (NFBC 1986 *v*. NFBC 1966) in respect to incidence of psychosis was 1.88 (95% CI 1.49–2.38), that indicated that members of NFBC 1986 had higher risk to develop psychosis compared with NFBC 1966. After adjustments with parental psychosis, place of residence, paternal age and education, the HR was 1.73 (95% CI 1.35–2.23) (not shown in tables).

### Sex differences in psychosis

The proportion of psychosis in men was significantly higher in the NFBC 1966 than in NFCB 1986, (*p* < 0.001). When only men with psychoses were compared between cohorts, the proportion of individuals with a secondary education was lower (*p* < 0.001) and rural residence was higher (*p* = 0.003) than in the NFBC 1966. In women with psychosis, only the proportion of individuals with a secondary education was lower in the NFBC 1966 (*p* < 0.001).

In NFBC 1986, the mean age of onset of psychosis was earlier in women than in men (20.15 years (s.d.: 3.88) *v*. 21.61 years (s.d.: 3.52); *p* = 0.01) (not shown in tables). Men had more schizophrenia (narrow) diagnoses (*p* = 0.013), (psychotic) major depression (*p* = 0.011) and other psychoses (*p* = 0.078) compared with men in the NFBC 1966. Also women in NFBC 1986 had more schizophrenia (*p* = 0.01) and more (psychotic) major depression (*p* = 0.003), but in addition also more (psychotic) bipolar disorder (*p* = 0.05) than women in the NFBC 1966 (c.f. Supplementary material 2). Especially up to age 18, women in the NFBC 1986 had the highest cumulative incidence of psychosis (see Fig. 1). Among female members of the NFBC 1986 with psychosis up to age 18 32% (*n* = 10) had schizophrenia (narrow), 13% (*n* = 4) had schizophrenia spectrum disorder, 6.5% (*n* = 2) had bipolar disorder (psychotic), 22.5% (*n* = 7) had major depression (psychotic), 10% (*n* = 3) brief psychosis and 16% (*n* = 5) had other non-organic psychoses (not shown in tables).

### Schizophrenia *v*. all other psychosis

Prevalence of risk factors was compared between subjects with schizophrenia diagnosis *v*. all other psychoses within cohorts. There were significantly more male individuals with schizophrenia than with all other psychoses in the NFBC 1986 (*p* = 0.045). However, no other statistical differences were observed (not shown in tables).

## Discussion

### Main findings

Even if the cumulative incidence of schizophrenia remained the same when the two cohorts, set up 20 years apart were compared, that for all psychoses increased (from 1.0 to 1.9%). Also the onset, based on first treatment or disability pension, had become earlier. The highest risk for early onset shifted from men to young women. Of the studied risk factors for psychosis, only parental psychosis was a significant risk factor of psychosis (HR ≥ 3.0) in both cohorts.

### Time trends in the incidence of schizophrenia

Studies from high-income countries across time are inconsistent, some reported a decline in the incidence of schizophrenia (range: −9 to −66%), no change or an increase in incidence rates (range: +18 to +64%) (Warner, 2004). The incidence of schizophrenia in Finland has declined in cohorts born between 1954 and 1965 (Suvisaari *et al*. 1999). In the 1980s, first admission rates due to schizophrenia decreased with only slightly increase thereafter (Salokangas *et al*. 2011). The life-time schizophrenia prevalence in an internal isolate from Northeastern Finland has been higher (2.2%) compared with other regions (1.2%) in Finland (Hovatta *et al*. 1997). In the present study, NFBCs represent mainly other northern regions. Thus, genetic predisposition, while being probably a strong contributing factor, might not entirely explain higher psychosis incidence in Northern Finland. Future research should explore gene environment interaction.

### Changes in risk factors in respect to incidence of psychosis

In the two NFBCs, the proportions of risk factors of psychosis had both increased as well as decreased within 20 years, e.g. rural residence earlier parental age and low maternal education were less frequent in the NFBC 1986. On the other hand, more infants with low birth weight <2500 g should have survived to adulthood due to improved quality of health care and it could increase the incidence of psychosis. The analysis showed that the difference in incidence of psychosis between the two cohorts was not dependent on change in prevalence of risk factors, since after the adjustment for potential risk factors the decrease in pooled HR (NFBC 1986 *v*. NFBC 1966) was only 8% (not presented in tables). Paternal age has not been associated with increased psychosis risk in either of the NFBCs, but in a meta-analysis – including also the NFBC 1966 – advanced paternal age was linked with psychosis risk (relative risk, RR = 1.65; 1.46–1.89; *p* < 0.01). The recent meta-analysis by Rasic *et al*. (2013) has shown that at age 20 and over offspring of parents with schizophrenia had 7.87 RR of schizophrenia (95% CI 4.14–14.94, *p* < 0.001), that is lower compared to previously found 13% risk (Gottesman, 1991). Also the recent follow-up of NFBC 1966 (age of 46) found that parental psychosis increased risk for any psychosis only with HR 2.9 (95% CI 1.90–4.89) (Keskinen, 2015).

In the present study only limited amount of early risk factors could be investigated. Data on, e.g. childhood adversities/stress were not collected and data for adolescence cannabis use were not available for NFBC 1966. In the 1990s, an increase in the number of psychiatric inpatients consuming illegal drugs was observed in Finland (Pirkola & Sohlman, 2005), but in the NFBC 1966 there were no cases of schizophrenia with a cannabis abuse (Koskinen *et al*. 2010). Later in the NFBC 1986, adolescent cannabis use was linked with prodromal symptoms of psychosis (Miettunen *et al*. 2008). Migration could also affect the risk of psychosis (Matheson *et al*. 2014), but the NFBCs have a homogeneous population with very low prevalence of non-Finnish parents.

### Diagnostic system changes

Diagnostic changes are a methodological challenge. During the total 40 years of the NFBCs, the diagnostic system has changed from ICD 8 via ICD 9 to ICD 10. During ICD 8 period Finnish psychiatrists followed the Bleulerian principles in diagnosing schizophrenia, i.e. preferring schizophrenia for manic depressive illness (Salokangas, 1985). In the time of the ICD 9, the diagnostic criteria for mental disorders were adopted with slight modification from DSM–III–R, which had narrower definition for schizophrenia and a requirement of 6-month duration of symptoms (Kuoppasalmi *et al*. 1989). Later, in the ICD 10 it is only 1 month as was the case also for schizophreniform disorder in ICD 8/9. Thus, schizophreniform disorder was included to schizophrenia (narrow) in the present study. Diagnostic practices have been assessed in the NFBC 1966, firstly up to 1993 and then up to 1997, showing that 43 and 48% of schizophrenia cases were diagnosed as ‘schizophreniform disorder’ and ‘other psychosis’ (Isohanni *et al*. 1997; Moilanen *et al*. 2003). In NFBC 1986, such studies have not been conducted. Even if this should not dramatically bias our results, the shift from schizophrenia to other types of psychoses might include some misclassifications due to the change in diagnostic practise over the period (Munk-Jorgensen, 1986; Allardyce *et al*. 2000; Sorvaniemi *et al*. 2006).

### Health system changes and new registers

Schizophrenia can be seen as a continuum of symptomatology (Van Os *et al*. 2009) and early detection of first-episode psychosis increases the chances of milder deficits and superior functioning (ten Velden Hegelstad *et al*. 2012). According to clinical staging model, earlier identification and referral of those at risk of psychosis in a non-stigmatising way (by e.g. school counsellors, primary/specialist care) play essential role in early prevention (Scott *et al*. 2013). As a result of the mental healthcare actions in Finland, outpatient mental health visits increased (from 1.6 million to 2.3 million) during 1997–2010 period (Pirkola & Sohlman, 2005). The admissions of adolescent patients more than doubled between 1991 and 2003 (Salokangas *et al*. 2011). Earlier psychosis detection due to more options in mental health services could partially explain higher rates of psychosis in the NFBC 1986 than in the NFBC 1966, but also the decrease of the most severe forms of psychosis. Differences in incidence and onset (NFBC 1986 *v*. NFBC 1966) may be explained, at least partially, by earlier help-seeking behaviour and detection in younger people, especially in those with affective symptoms, which may be considered as a prodromal stage of psychosis. That also could explain higher incidence among females.

Early screening should result in decreased duration of untreated psychosis (DUP), but the time trends of DUP among adolescents have not been studied and no large-scale prevention programmes have been conducted among them in Northern Finland. A review by Singh (2007) observed that in 13 selected studies from high-income countries mean DUP varied from 25 to 722.8 months. In the NFBC 1966, DUP was 33.02 weeks (Penttilä *et al*. 2010), which is shorter compared with almost all studies in this review. However, there is no evidence to state that DUP in NFBC 1986 is shorter than in NFBC 1966. Thus, health system changes could have an effect on incidence of psychosis in Northern Finland, but this requires further research.

The new registers available in the NFBC 1986 (*v*. NFBC 1966) could have increased the number of identified cases. However, the proportion of outpatients was low and fully covered by SII registries in the NFBC 1966. Due to deinstitutionalisation, the out-patients registers play more important role for cases identification in NFBC 1986 than in the NFBC 1966, but should not have had any major effect on the results. Parental psychosis data were available only since 1962 and it could result in underestimation of parental psychosis in NFBC 1966.

### Strengths

The use of two cohorts set up 20 years apart covering the same geographic region is the main strength allowing greater comparability of two cohorts. The prospective data collection from two successive subjects’ generations (Rantakallio, 1988) and the use of Finnish national registers with good validity and high coverage (Sund, 2012) are the other strengths. The birth cohort design allowed us to study very early as well as some prospectively collected risk factors.

### Limitations

The comparison of the two cohorts only up to the age 27 years based on the follow-up available in NFBC 1986. In the latest NFBC 1966 follow-up in 2012 (age 46 years), the incidence of psychosis was 3.1 and 46.5% among diagnosed had schizophrenia, 13.1% had bipolar disorder with psychotic symptoms and 33.1% had depression with psychotic symptoms (Keskinen, 2015). Still, when comparing the two cohorts until the age 27, the peak risk period for developing a psychosis (adolescence) is included (Paus *et al*. 2008). The samples include only help-seeking/treated cases and the results could at least partially be influenced by trends in diagnostic practise and the mental health services reorganisation that took place in Finland. It might be that affective psychoses and non-schizophrenia cases were under-diagnosed and untreated in the NFBC 1966 even though their incidence in early adulthood may be low (Mäki *et al*. 2010), while earlier detection of cases could have been possible in the NFBC 1986. Thus, some of the results are based on few cases. Still, the quality of Finnish register is high and according to a study by Perälä *et al*. (2007) the underestimation of cases was only 25% in CRHC and 13.7% in all other registers. Lastly, non-studied or not collected risk factors may have also affected the incidence of psychosis at least in some extent.

In conclusion, comparison of the two birth cohorts within 20 years covering altogether a period of 40 years showed increase in incidence of psychosis in younger NFBC 1986. Shift in types of diagnosis, to less severe psychoses, and to earlier onset, especially among women, were found. Studied risk factors did not explain the time trends of psychosis. The observed differences could be due to real changes in incidence or they can be attributable to changes in diagnostic practices, or to early psychosis detection and treatment. However, more research is needed to explore reasons for these changes.

## Supplementary material

For supplementary material accompanying this paper visit http://dx.doi.org/10.1017/S2045796016000123. click here to view supplementary material
