# Supplementary material for: A comparison of the cumulative incidence and early risk factors for psychotic disorder in young adults in the Northern Finland Birth Cohorts 1966 and 1986
Source: Epidemiol Psychiatr Sci. 2016 Mar 28;26(3):314–24. doi: 10.1017/S2045796016000123 (PMC6998683; doi:10.1017/S2045796016000123)
Supplement: Supplementary file 1 [file S2045796016000123sup.zip › S2045796016000123sup002.pdf]

Supplementary material 2 Psychoses in males and females in NFBC 1966[a] and 1986[b]

	Males			Females		
**Predictor**	**NFBC1966 (n=68)**	**NFBC 1986 (n=96)**	**p-value**	**NFBC 1966 (n=50)**	**NFBC 1986 (n=81)**	**p-value[c]**
Parental psychosis (until age 27)						
- no	59/68 (86.8%)	81/96 (84.4%)	0.823	39/50 (78.0%)	74/81 (91.4%)	0.031
- yes	9/68 (13.2%)	15/96 (15.6%)		11/50 (22.0%)	7/81(8.6%)	
Mother's education at birth						
- basic education	46/68 (67.6%)	22 /77 (28.6%)		34/49 (69.4%)	19/66(28.8%)	
- secondary education	19/68 (27.9%)	48/77 (62.3%)	<0.001	13/49 (26.5%)	43/66 (65.2%)	<0.001
- higher education	3/68 (4.4%)	7/77 (9.1%)		2/49 (4.1%)	4/66 (6.1%)	
Place of residence						
- urban	18/68 (26.5%)	47/93 (50.5%)		20/50(40.0%)	35/78 (44.9%)	
- rural	50/68 (73.5%)	46/93 (49.5%)	0.002	30/50(60.0%)	43/78 (55.1%)	0.587
Maternal age						
- <20	7/67 (10.4%)	6/96 (6.3%)		3/50(6.0%)	3/81 (3.7%)	
- 20-35	45/67 (67.2%)	75/96 (78.1%)	0.287	38/50(76.0%)	68/81 (84.0%)	0.511
- >35	15/67 (22.4%)	15/96 (15.6%)		9/50 (18.0%)	10/81(12.3%)	
Paternal age						
- <25	11/64 (17.2%)	20/95 (21.1%)		8/48 (16.7%)	8/81 (9.9%)	
- 25-40	45/64 (70.3%)	67/95 (70.5%)	0.630	36/48 (75.0%)	67/81 (82.7%)	0.502
- >40	8/64 (12.5%)	8/95 (8.4%)		4/48 (8.3%)	6/81 (7.4%)	
Diagnostic subcategories						
- Schizophrenia narrow	32/68 (47.1%)	27/96 (28.1%)	0.013	17/50(34.0%)	12/81(14.8%)	0.010
- Schizophrenia spectrum[d]	7/68 (10.3%)	6/96 (6.3%)	0.345	5/50 (10.0%)	5/81 (6.2%)	0.505
- Bipolar disorder with psychotic features	4/68 (5.9%)	7/96 (7.3%)	0.999	1/50 (2.0%)	10/81 (12.3%)	0.051
- Major depressive episode with psychotic features	0/68 (0%)	9/96 (9.4%)	0.011	2/50 (4.0%)	19/81 (23.5%)	0.003
- Brief psychosis	9/68 (13.2%)	12/96 (12.5%)	0.890	4/50 (8.0%)	8/81 (9.9%)	0.999
- Other psychoses	16/68 (23.5%)	35/96 (36.5%)	0.078	21/50 (42.0%)	27/81 (33.3%)	0.317

Onset age (mean/SD)	21.38 (3.12)	21.61 (3.53)	0.670	21.74 (3.36)	20.15 (3.88)	0.018

[a]NFBC 1966 =11621, [b]NFBC 1986 =9329, [c]p-values are from chi-square and t-test, SD=standard deviation, [d] schizophrenia spectrum includes schizoaffective disorder and delusional disorder
