# Supplementary figures and images for: A comparison of the cumulative incidence and early risk factors for psychotic disorder in young adults in the Northern Finland Birth Cohorts 1966 and 1986
Source: Epidemiol Psychiatr Sci. 2016 Mar 28;26(3):314–24. doi: 10.1017/S2045796016000123 (PMC6998683; doi:10.1017/S2045796016000123)

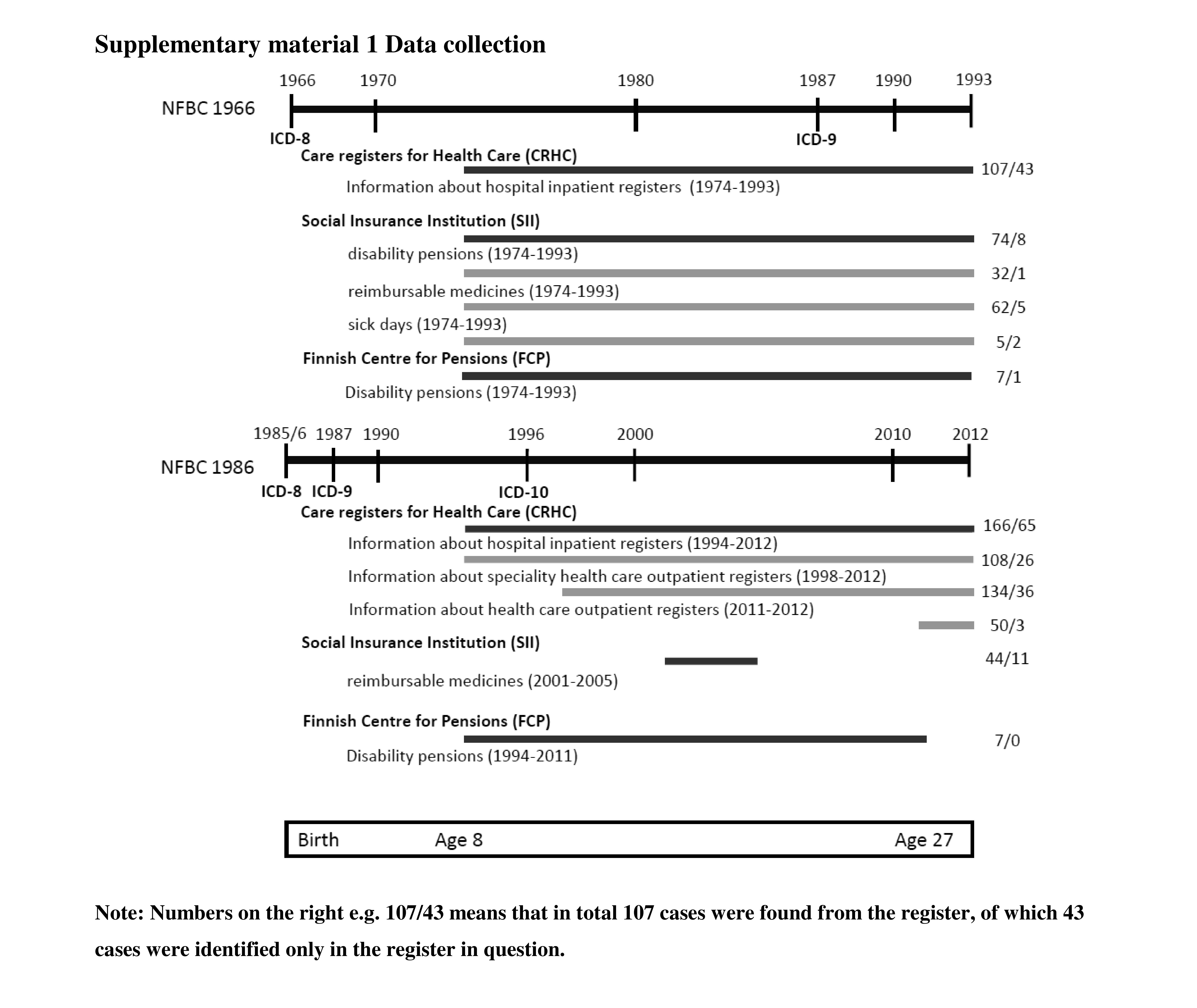

Supplement: Supplementary file 1 [file S2045796016000123sup.zip › S2045796016000123sup001.jpg]
